# Magnetic Behavior in TiS_3_ Nanoribbon

**DOI:** 10.3390/ma12213501

**Published:** 2019-10-25

**Authors:** Shengqiang Lai, Yongping Du

**Affiliations:** 1 The School of Physics, Nanjing University, Nanjing 210093, China; lai@smail.nju.edu.cn; 2 Department of Applied Physics and Institution of Energy and Microstructure, Nanjing University of Science and Technology, Nanjing 210094, China

**Keywords:** TiS_3_, nanoribbon, magnetic property, strain effect

## Abstract

The electronic structure, magnetic properties and strain response of N-a-TiS_3_ nanoribbons are investigated by first-principles calculations. We find that the magnetic ground state is strongly dependent on width of **a**-TiS_3_. When N equals an odd number the ground state is a ferromagnetic (FM) metal, meanwhile, when N equals an even number the ground state is an anti-ferromagnetic (AFM) metal. More interestingly, a tensile strain as large as 6% can tune the 9-**a**-TiS_3_ nanoribbon from a FM metal to a half metal. A 4% tensile strain also causes a phase transition from AFM to FM ground state for 10-**a**-TiS_3_ nanoribbon. Our findings show that N-**a**-TiS_3_ is a promising candidate for spintronic and electronic applications.

## 1. Introduction

The properties of materials are essentially associated with dimensionality. Due to low dimensionality, quantum confinement and their promising applications in spintronics, optronics, thermoelectrics [1,2,3,4], etc., one-dimensional (1D) nanostructures, such as nanotubes, nanowires, nanobelts, and nanoribbons, have drawn a lot of attention during the past two decades [5,6,7]. When graphene is cut into 1D graphene nanoribbons, even more fantastic properties are predicted and some have already been verified experimentally [8,9,10,11]. Especially, the zigzag graphene nanoribbons (ZGNRs) were predicted to be antiferromagnetic semiconductors [12,13]. More interestingly, many theoretical studies have demonstrated that the half-metallicity in ZGNRs can be realized under external transverse electric fields [14,15], or by selective chemical modifications [16,17,18]. Many other methods, like doping [17] or defects [19], were applied to tune or control the magnetism in ZGNRs. Moreover, ZGNRs can carry a spin current response to an external electric field, which opens a new path to the application of spintronics [14]. In addition to ZGNRs, magnetism hasalso been predicted in many other nanoribbons, such as CoTe [1], Fe_2_GeAl [2], MnSi [3], BN [20], MoS_2_ [21], black and blue phosphorus [22,23], andZnO [24].

Very recently, a new two-dimensional (2D) material, namely TiS_3_, has been successfully synthesized [25,26,27,28]. It is reported that monolayer TiS_3_exhibits a direct band gap of 1.1–1.2 eV [29], making the bandgap of TiS_3_ comparable to that of silicon (1.1 eV). Also, numerical results show that the bandgap of TiS_3_ is quite robust, almost independent of layer thickness, vertical strain and stacking order [30]. Moreover, a recent theoretical study proposed that TiS_3_ is expected to have a higher electron mobility of 10,000 cm^2^V^−1^s^−1^ [29]. Such a robust bandgap and ultrahigh electron mobility make TiS_3_ a good candidate for nanoelectronics and optoelectronics application. The elastic modulus, carrier mobility and band structure of TiS_3_ are strongly anisotropic due to its highly anisotropic crystal. Experimentally, nanoribbons of TiS_3_ were successfully prepared [25,26,27,28]. Although, there are several studies on nanoribbons of TiS_3_ [25,26,27,28,31,32,33], the magnetism of TiS_3_ nanoribbon still lacks systematic study.

In this article, using first-principles calculation, we systematically study the magnetic properties of TiS_3_ nanoribbon. Our results show that **a**-TiS_3_ NR is a spin-polarized metal with local magnetic moments at the edge, while **b**-TiS_3_ NR is nonmagnetic semiconductor. More interestingly, the magnetic ground state of N-**a**-TiS_3_ NRs (N = 7, 8, 9, 10, 11) are width dependent. When N is odd, the ground state is ferromagnetism (FM), meanwhile the ground state is anti-ferromagnetism (AFM)when N is even. This magnetic behavior is very different from other magnetic nanoribbons, such as zigzag black phosphorene nanoribbons [22] and zigzag graphene nanoribbons [12,13], whose magnetic properties are almost independent on the width of nanoribbon. We also find that compressive strain almost does not change the magnetic and electric properties of **a**-TiS_3_ NRs. However, according to our calculations in 9-**a**-TiS_3_ NR and 10-**a**-TiS_3_, tensile strain can change the magnetic and electric properties dramatically. 9-**a**-TiS_3_ NR becomes a half metal under a tensile as large as 6%, and the AFM ground state of 10-**a**-TiS_3_ is tuned into a FM ground state at about 4% tensile strain. Our findings show that the a-TiS_3_ NRs are promising candidates for spintronics and electronics application.

## 2. Methods

All the calculations were carried with Vienna ab initio Simulation Package (VASP, version 5.3.2, University of Vienna, Vienna, Austria) [34,35]. The frozen-core projector augmented wave (PAW) method and the generalized gradient approximation (GGA) of Perdew–Burke–Ernzerhof (PBE) [36] were adopted. Cutoff energy was set as 500 eV for plane-wave expansion of the electronic wave function, and appropriate k-point meshes (9 × 1 × 1) were used for geometric optimization and self-consistent calculation. All structures were relaxed until the force on each atom was less than 0.01 eV/ Å. The energy convergence criteria were set to 1.0 × 10^−6^ eV. A vacuum spacing of 15 Å was used so that the interaction in the non-periodic directions can be neglected. Since both Ti and S are not heavy elements, spin-orbit coupling (SOC) is expected to be small, thus the spin-orbit interaction is not included in all calculations.

## 3. Results and Discussion

The unit cell of monolayer TiS_3_ is a rectangle. Our optimized lattice constants are a = 5.02 Å, b = 3.40 Å which are in good agreement with previous experimental result (4.96 and 3.40 Å) [37], and theoretical result (5.02 and 3.41 Å) [31]. There are two particular ways to cut monolayer TiS_3_ into nanoribbons (cutting along the **a** axis or **b** axis). Then, two types of nanoribbons are indicated as N-**a**-TiS_3_ NR and N-**b**-TiS_3_ NR, where N indicates the number of Ti atoms in the unit cell of ribbon and **a**-TiS_3_ NR and **b**-TiS_3_ NR are along the **a** and **b** lattice vectors, respectively. Our calculations show that N-**b**-TiS_3_ NRs are semiconductors without any local magnetic moment, which is consistent with previous theoretical results [31]. Thus, in the following discussion we will eliminate the N-**b**-TiS_3_ NRs and mainly consider the N-**a**-TiS_3_ NRs.

**Figure 1:**
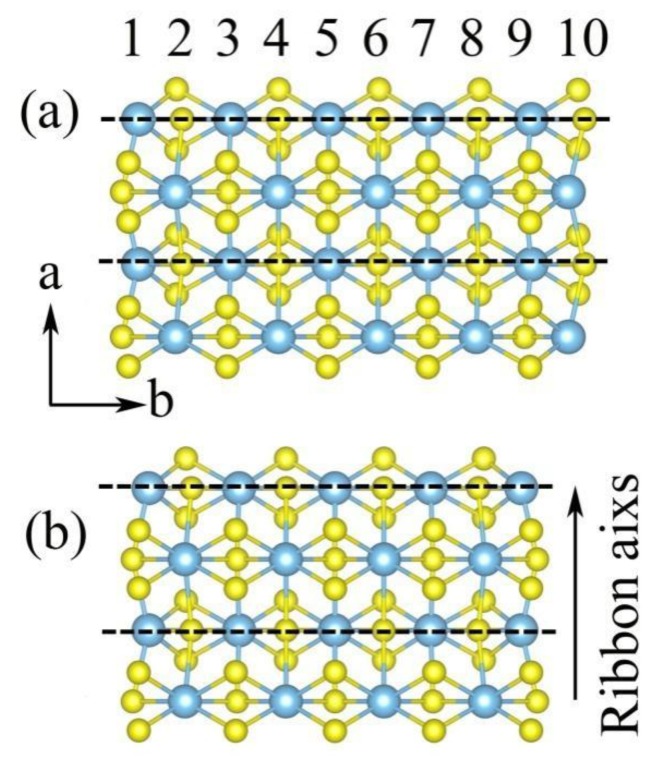
(**a**) The crystal structure of 10-**a**-TiS_3_nanoribbon (NR), and; (**b**) the crystal structure of 9-**a**-TiS_3_. Two dashed lines indicate the unit cell of nanoribbon along the a lattice vector. The dashed lines indicate the TiS_3_ chain in monolayer TiS_3_.

Here, we take N = 7–11 for a-TiS_3_ NRs. Figure 1 shows the crystal structure of 10-**a**-TiS_3_ NR and 9-**a**-TiS_3_ NR. In particular, edge atoms may influence the physical properties. For **a**-TiS_3_ NR, the edge atoms are S and Ti with dangling bonds which are local states around Fermi level as shown later. However, there are some differences in crystal structure between 9-**a**-TiS_3_ NR and 10-**a**-TiS_3_ NR. Dashed lines in Figure 1a,b also indicate the TiS_3_ chain in monolayer TiS_3_. In 9-**a**-TiS_3_ NR, the terminal atoms for every TiS_3_ chain are the same, namely both of two edge atoms are Ti or S atoms for the same chain as shown in Figure 1b. However, in 10-**a**-TiS_3_ NR, the edge atoms for every TiS_3_ chain are different, i.e., one terminal atom is Ti and the other is an S atom shown in Figure 1a. Moreover, for N equal to all other odd numbers (or even numbers) the edge atom arrangement is the same as the N = 9 (or 10). This different edge atom arrangement will cause a different magnetic ground state of two NRs as discussed below.

**Figure 2:**
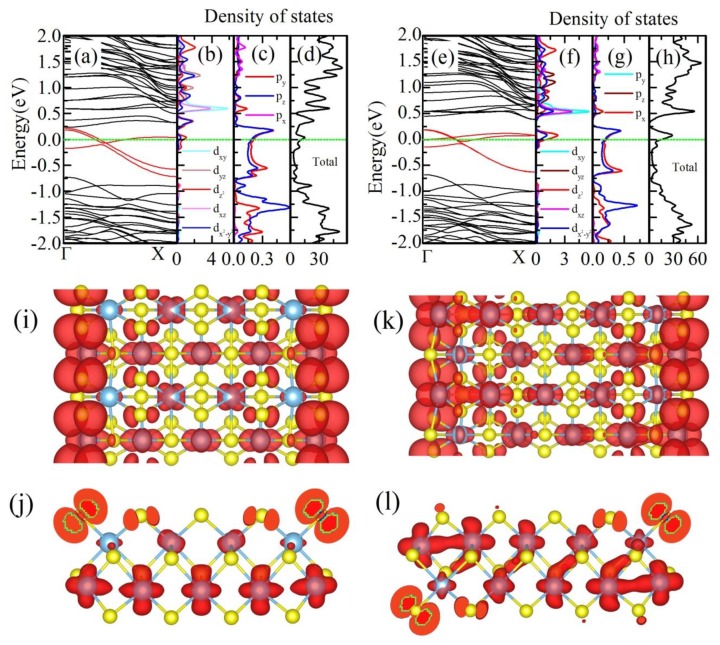
(**a**) and (**e**) are the band structures of 9-**a**-TiS_3_ NR and 10-a-TiS_3_, respectively, within the GGA calculation. Three bands, which cross the Fermi level and are denoted by red color, are the edge states. (**b**) and (**f**) are the density of states of d-orbital of edge Ti atoms in 9-**a**-TiS_3_ NR and 10-a-TiS_3_ NR respectively. (**c**) and (**g**) are the density of states of p-states of edge S atoms in 9-**a**-TiS_3_ and 10-**a**-TiS_3_ NRs; (**d**) and (**h**) are the total density of states of 9-**a**-TiS_3_ and 10-**a**-TiS_3_ NRs respectively. (**i**) and (**j**) are the charge density of three red bands of 9-**a**-TiS_3_ NR. (**k**) and (**l**) are the charge density of three red bands of 10-**a**-TiS_3_ NR. (**i**) and (**k**) are the top view while (**j**) and (**l**) are the side view along the period direction.

Firstly, we calculate the electronic structure of 9-**a**-TiS_3_ NR and 10-**a**-TiS_3_ NR under GGA approximation without any spin polarization. The electronic structures of two nanoribbons are shown Figure 2. Three bands, denoted by red color, cross the Fermi energy indicating the metallic behavior of **a**-TiS_3_ NR (shown in Figure 2a,e). Further analysis shows that these three bands are the edge states. Density of states (DOS), shown in Figure 2, indicate that three bands crossing the Fermi level are mainly contributed by the unbonding $p_{y}$ and $p_{z}$ states of edge S atoms and $d_{z^{2}}$ and $d_{x^{2}-y^{2}}$ of edge Ti atoms. A peak of DOS near the Fermi energy, as shown in total DOS, indicates the possibility of spin polarization driven by Stoner instability [38].

To clarify the effect of edge atoms, we plot the charge density isosurfaces of these three bands which cross the Fermi energy and the results are shown in Figure 2i–l. These pictures clearly illustrate the charge density is mainly localized at edge S and Ti atoms which again proves that three bands denoted by red lines are indeed the edge states. As we discussed before, the arrangement of edge atoms is different between 9-**a**-TiS_3_ NR and 10-a-TiS_3_ NR. Thus, charge distribution is different for these two **a**-TiS_3_ NRs. In 9-**a**-TiS_3_ NR, the charges are mainly localized at edge S atoms which are in the TiS_3_ chain, whose the end atoms are both sulfur atoms, shown in Figure 2i,j. The p orbital shape of charge density on edge S atoms are the hybridization of $p_{y}$ and $p_{z}$ unbonding state. However, the contribution of Ti-d state in TiS_3_ chain with two end Ti atoms is more complicated. The charge distribution shows that all Ti atoms in this chain have almost equal contributions to the three red bands. This means that the hybridization between Ti atoms is very strong. However, there is a very different charge distribution in 10-**a**-TiS_3_ NR. As we discussed previously, the terminals of TiS_3_ chain were one S atom and one Ti atom, rather than the two same atoms. Thus, the charge distribution of 10-**a**-TiS_3_ NR is very different from that of 9-**a**-TiS_3_ NR. In Figure 2k,l, we can see that there are p orbital shapes of charge density around edge S atoms, as in 9-**a**-TiS_3_ NR. More interestingly, the charge distribution on Ti atoms in the same chain are not equal at all. The biggest contribution comes from the edge Ti atom, and then the charge density decreases from the edge Ti atom to other side of NR which is different from the situation in 9-**a**-TiS_3_ NR. As we discuss later, this difference would cause the different magnetic ground states.

The previous theoretical study has proposed that the **a**-TiS_3_ NRs have local magnetic moment [31]. But detailed studies of magnetic properties are still lacking. Thus, we perform the spin-polarized calculation to find the magnetic properties of N-**a**-TiS_3_ NRs.

**Figure 3:**
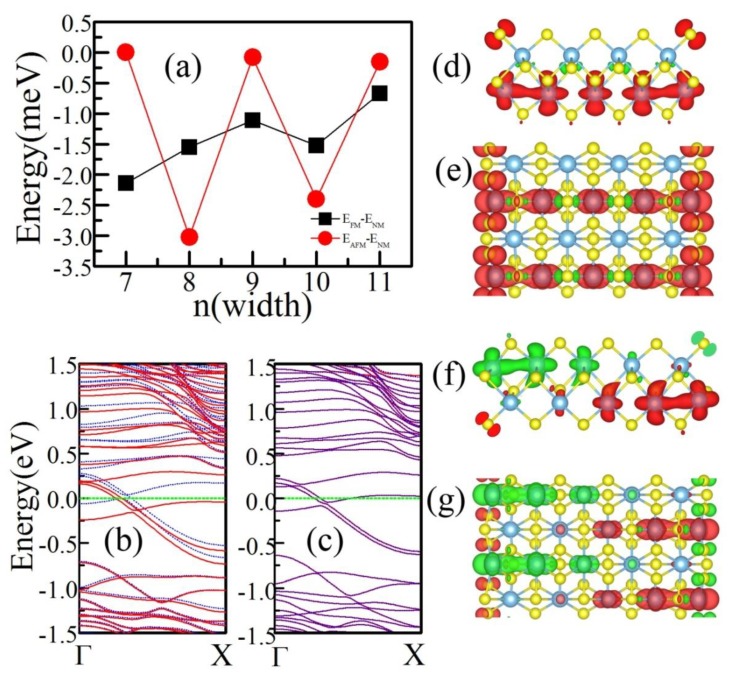
(**a**) The energy of magnetic ground state changes as the width of **a**-TiS_3_ NR. (**b**) The spin-polarized band structure of the FM ground state of 9-**a**-TiS_3_ NR, red lines (blue dashed lines) indicate spin up (down) component. (**c**) The spin-polarized band structure of AFM ground state of 10-**a**-TiS_3_, red lines (blue dashed lines) denote spin up (down) component. (**d**) and (**e**) are spin density of FM state of 9-**a**-TiS_3_ NR, (**d**) is the side view from the period direction of **a**-TiS_3_ NR and (**e**) is the top view. (**f**) and (**g**) are the spin density of the AFM state of 10-**a**-TiS_3_ NR, (**f**) is side view from the period direction of 10-**a**-TiS_3_ NR and (**g**) is the top view.

To explore the magnetic ground state of N-**a**-TiS_3_ NRs, we set two magnetic configurations, one is a FM arrangement with all magnetic moments in the same direction and the other is an AFM configuration with the magnetic moments of the two edges anti-parallel. From the results of the total energy calculation, we can easily find that the ground state of N-**a**-TiS_3_ NRs are not all FM which is different from the previous theoretical results [31]. The magnetic ground state varies as the width changes. Figure 3a shows that the energy difference between the magnetic state and nonmagnetic state, one can easily see that when N is odd, the ground state is FM, meanwhile N is even, the ground state is AFM. This width-dependent magnetic ground state is different from other 2D material NRs like black phosphorus [23].

Our calculations also reveal that all N-**a**-TiS_3_ NRs have metallic behavior with bands crossing the Fermi level. The electronic band dispersion of ground magnetic states of 9-**a**-TiS_3_ NR and 10-**a**-TiS_3_NR are shown in Figure 3b,c, respectively. For FM ground state of 9-**a**-TiS_3_, there is a spin split near the Fermi level. The total magnetic moment of the unit cell is 0.86 μB which is consistent with the previous calculation [31]. Due to the AFM ground state, two spin components of 10-**a**-TiS_3_ are exactly overlapped indicating a perfect AFM state. We also checked the effect of SOC, and we found that the SOC does not change the magnetic ground states of N-a-TiS3 NRs, due to the small SOC effect in light elements.

To get further insight into the spin polarization in **a**-TiS_3_ NRs, we also calculated the spin density, which is the difference between spin-up and spin-down channels, and show them in Figure 3d–g. Figure 3d,e are the spin density of FM 9-**a**-TiS_3_ NR. The magnetic moment is mainly located on edge S and edge Ti atoms; in addition, the magnetic moment is still located in Ti atoms in the TiS_3_ chain whose terminal atoms are Ti atom. The magnetic moment decreases from 0.144 μB (edge Ti atom) to 0.131 μB (center Ti atom). The distribution of magnetic moment also indicates the strong hybridization between Ti atoms. Figure 3f,g are the spin density of 10-**a**-TiS_3_ NR in AFM ground state. The magnetic moment is mainly located at edge S atoms and Ti atoms. Due to the strong hybridization between Ti atoms in the same TiS_3_ chain, magnetic moments turn to zero gradually from the terminal Ti to center one.

**Figure 4:**
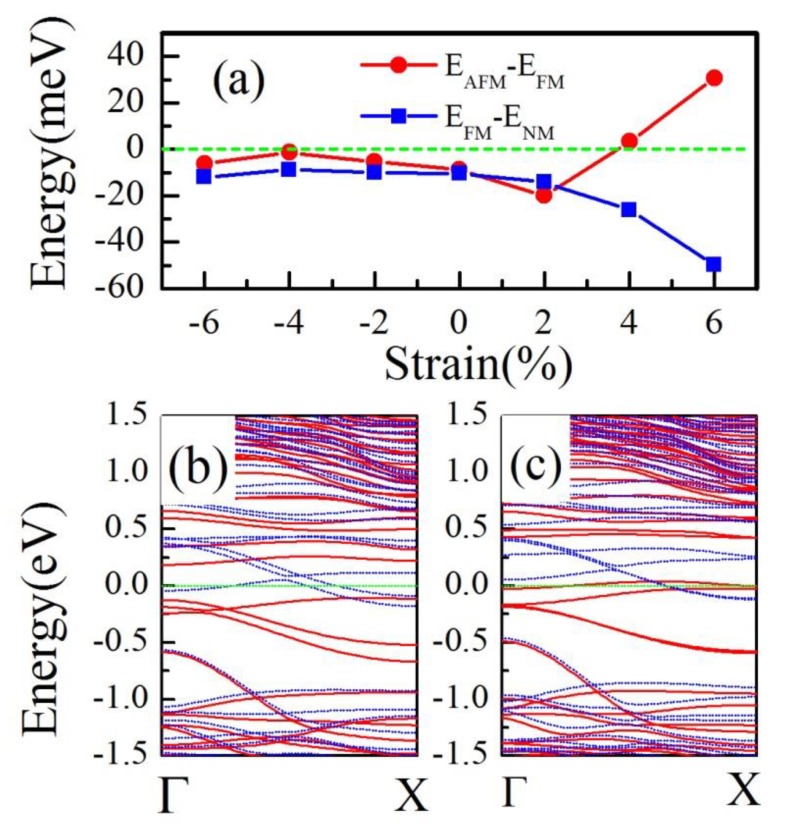
(**a**) Energy difference between nonmagnetic (E_NM_)/anti-ferromagnetic (E_AFM_) and ferromagnetic (E_FM_) as a function of strain. (**b**) The spin-polarized band structure of 9-**a**-TiS_3_ NR under 6% tensile strain. (**c**) The spin-polarized band structure of 10-**a**-TiS_3_ NR under 6% tensile strain.

As is well known, the edge states usually are sensitive to strain, doping, and the external field. Here we focused on the effect of strain on the edge states. A uniaxial strain varying from −6% to 6% was applied to investigate the magnetic and electronic properties. The energy differences between FM and nonmagnetic/AFM states of 9-a-TiS_3_ NR (10-a-TiS_3_ NR) are shown in Figure 4a. We found that for both 9 and 10-a-TiS_3_ NR, the compressive strains have a small effect on the magnetic ground state. Although the FM ground state is not changed by tensile strain, the electronic structure had a dramatic change. We calculated the band structure of 9-a-TiS_3_ NR under the various strains, and found that when a tensile strain as large as 6%, FM 9-a-TiS_3_ NR become a half metal. The band structure of 9-a-TiS_3_ NR under 6% tensile strain is plotted in Figure 4b with the red lines denoting the spin up component and blue dashed lines denoting the spin down component. We can clearly see that the spin-up component opens an indirect band gap about 0.2 eV while three spin-down bands cross Fermi energy indicating metal property. The energy difference between AFM and FM show that there is an AFM-FM phase transition of 10-a-TiS_3_ NR under a 4% tensile strain. We also plotted the band structure of 10-a-TiS_3_ NR under the 6% tensile strain, a large spin-split around Fermi energy shows the FM property.

## 4. Conclusions

In summary, we investigated the electronic and magnetic properties of TiS_3_ NRs and strain effect by first-principles calculations. Our results reveal that b-TiS_3_ NRs are non-magnetic semiconductors consistent with previous results. However, the magnetic ground states of N-a-TiS_3_ NRs are strongly dependent on the width of NRs. When N is an (even) odd number, N-a-TiS_3_ NRs are (anti-)ferromagnetic metals. The strain effect is also investigated. The compressive strain has little effect on the electronic and magnetic properties of 9(10)-a-TiS_3_ NRs. On the contrary, a 6% tensile strain will tune 9-a-TiS_3_ NR from a FM metal into a half metal while a 4% tensile strain cause a AFM-FM phase transition of 10-a-TiS_3_ NR. Our findings make the N-a-TiS_3_ NRs promising spintronic devices.
